# Showing Value in Newborn Screening: Challenges in Quantifying the Effectiveness and Cost-Effectiveness of Early Detection of Phenylketonuria and Cystic Fibrosis

**DOI:** 10.3390/healthcare3041133

**Published:** 2015-11-11

**Authors:** Scott D. Grosse

**Affiliations:** National Center on Birth Defects and Developmental Disabilities, Centers for Disease Control and Prevention, Atlanta, GA 30341, USA; E-Mail: sgrosse@cdc.gov; Tel.: +1-404-498-3074

**Keywords:** health economics, cost-benefit, cost-effectiveness, genetic testing, neonatal screening, cystic fibrosis, phenylketonuria

## Abstract

Decision makers sometimes request information on the cost savings, cost-effectiveness, or cost-benefit of public health programs. In practice, quantifying the health and economic benefits of population-level screening programs such as newborn screening (NBS) is challenging. It requires that one specify the frequencies of health outcomes and events, such as hospitalizations, for a cohort of children with a given condition under two different scenarios—with or without NBS. Such analyses also assume that everything else, including treatments, is the same between groups. Lack of comparable data for representative screened and unscreened cohorts that are exposed to the same treatments following diagnosis can result in either under- or over-statement of differences. Accordingly, the benefits of early detection may be understated or overstated. This paper illustrates these common problems through a review of past economic evaluations of screening for two historically significant conditions, phenylketonuria and cystic fibrosis. In both examples qualitative judgments about the value of prompt identification and early treatment to an affected child were more influential than specific numerical estimates of lives or costs saved.

## 1. Introduction

Newborn screening (NBS) to identify congenital disorders is a major public health success, saving lives and preventing disability in thousands of infants each year. Public health NBS programs in all higher-income countries organize the collection of dried blood spot (DBS) specimens on filter paper cards, have them tested in officially designated screening laboratories, and report the results back to health care providers [1,2]. NBS programs include short-term follow-up activities to ensure that children who do not pass screening receive appropriate diagnostic services; some programs go further and monitor long-term follow-up. NBS programs constitute the largest and most widespread public health genomics programs, although not all NBS disorders are primarily genetic in etiology [3,4]. In addition, point-of-care screening of newborns for conditions such as congenital sensorineural hearing loss and critical congenital heart defects may be mandated or promoted by legislation or regulation, and public health programs can support screening and follow-up with technical assistance and data systems [5,6].

Governments add new disorders to NBS panels because they believe that doing so provides good value. The first dimension of “value” in health care is the health benefit or clinical utility, *i.e.*, better outcomes for affected individuals. In health care, “value” is also commonly interpreted as the relative balance of health benefit and economic cost [7,8]. This paper suggests that although both dimensions of value are important, evidence of effectiveness is of primary importance. In addition, a broader definition of “value” encompasses all outcomes that are important to patients and their families, which includes the personal and diagnostic utility of genomic information—both benefits and harms—as well as the perceived quality of care received [9,10].

Commonly used criteria for deciding on NBS expansions include the magnitude of health benefits and the feasibility and costs of screening, diagnosis, and treatment [11,12,13,14]. One of the classical Wilson and Jungner screening criteria is that “The costs of case-finding (including diagnosis and treatment of patients diagnosed) should be economically balanced in relation to possible expenditure on medical care as a whole [15].” NBS decision makers typically consider cost and benefit as discrete criteria to be weighed qualitatively. Some decision-making bodies go further and explicitly consider the magnitude of benefits relative to costs using economic evaluations, e.g., cost-effectiveness [16]. Historically, however, most decisions on NBS expansions in the United States and Europe have not been based on economic criteria [16,17].

The primary focus of this paper is on how different epidemiologic methods and choice of data sources can lead to quite different estimates of the net health outcomes of NBS. Such disparate estimates in turn can lead to different conclusions regarding the magnitude of cost savings or cost-effectiveness. It is crucially important to assure that estimates of the effects of early identification are not confounded by changes in treatment patterns or differences in the representativeness of screened and clinically detected cohorts. Assessing outcomes is complex, and the reality is often more nuanced than the simple conclusions sought by policy makers.

This paper uses past policy decisions to screen for two historically significant conditions: phenylketonuria (PKU) and cystic fibrosis (CF). PKU was the first metabolic condition to be screened for in newborns, and decisions about initial implementation in the 1960s continue to influence NBS programs around the world. CF is the only NBS condition to be subjected to a large-scale randomized, controlled study of the effectiveness of screening and early treatment [13]. These salient case studies illustrate the empirical challenges in estimating effectiveness and cost-effectiveness in NBS.

### 1.1. Economic Evaluation Overview

The balance of benefits relative to costs of an intervention or policy can be quantified using either cost-effectiveness analysis (CEA) or cost-benefit analysis (CBA). In CBA health outcomes are expressed in terms of monetary values that are intended to represent the lost societal welfare from premature death or incapacitation whereas in CEA health outcomes are calculated separately from costs. A CEA compares multiple interventions in terms of total outcomes and total costs. An intervention that both costs less and has better outcomes than the next best alternative is said to be cost-saving or dominant, and one that has better outcomes but the additional cost is relatively moderate is considered cost-effective.

It should be noted that cost-effectiveness is not an absolute attribute of an intervention but depends on the comparator. A strategy may be cost-effective in one setting compared with one alternative but not cost-effective in a different context or faced with a different comparator. Economic evaluations are based on the economics principle of the counterfactual, which means that everything, other than the specific intervention being evaluated, is held constant [18].

The first distinction among economic evaluation methods is whether the analysis models health outcomes (reduced deaths or disease) or just numbers of cases detected. Calculating the cost to detect a case tells one nothing about the value of detecting and treating the disease in question and hence is not informative of the balance of costs and outcomes. Partial CEA studies only consider costs and numbers of cases detected, whereas full CEAs calculate both incremental costs and health outcomes [19].

Full CEAs report whether an intervention is either cost-saving, or cost-effective, with an incremental cost-effectiveness ratio (ICER) that is considered favorable. Full CEAs are of two types: those that use “natural” measures of health, such as life-years saved, and those that use summary measures of health such as the quality-adjusted life-year or QALY [20]. CEAs that calculate incremental cost per QALY are also referred to as cost-utility analyses because QALYs are calculated using health utility (or health-related quality of life) scores for health states on a scale of 0 to 1 where 0 represents death and 1 perfect health [21]. Calculating QALYs for pediatric interventions such as NBS is particularly challenging [22,23,24].

Whether a given ICER is considered to provide good value depends on the decision maker. Decision-making bodies in some countries set threshold values for ICERs or use benchmarks as rough guides to value, particularly in the arena of pharmaceutical coverage decisions. Examples of popular but arbitrary ICER thresholds include $50,000, £29,000, or €30–40,000 per QALY [25,26,27]. Alternatives to a single threshold include a range of values, e.g., $50,000 and $250,000 per QALY as lower and upper bounds for cost-effectiveness. The World Health Organization has endorsed lower and upper bounds of 1 and 3 times a country’s per capita gross domestic product. However, the “revealed preferences” of decision makers in healthcare policy show that interventions with very high ICERs may be considered acceptable if the absolute expenditures are not too high. In particular, covered treatments for rare diseases, including NBS conditions, may exceed $1 million per QALY gained [28]. 

Many policy makers outside of medicine prefer CBA estimates expressed in terms of money and which allow for comparison across sectors such as health, environment, and transportation. Cost-benefit analyses in the medical arena often rely on the traditional “human capital” approach to estimating the monetary value of avoided death or disability as the discounted present value of the stream of future annual earnings or productivity. That approach is problematic. First, it ignores the costs of creating and maintaining the stock of human capital [29]. Second, some economists have advocated a “friction cost” approach to valuing the loss of a worker as the temporary cost of recruiting and training a replacement; that approach assumes that a child death involves no loss of future productivity [30]. Third, the human capital approach is inconsistent with the welfare theoretic basis of modern CBA, also referred to as benefit-cost analysis (BCA). In particular, it excludes the value that society places on avoidance of pain and suffering and the spillover effects of death and disability on other people. Exclusion of such valuations understates the economic value to society of disease prevention.

Since the 1970s, the dominant approach to CBA/BCA outside of health care involves assessments of consumer “willingness to pay” (WTP) to value health outcomes. More precisely, researchers assess individual WTP to reduce by a small amount the risk of adverse outcomes and aggregate across individuals to value the prevention of those outcomes [31]. Researchers use either stated preference methods (survey data) or revealed preferences from real-world behavior; the latter includes estimates of compensating wage differentials in relation to occupational fatality risks to estimate what is referred to in the United States as the “value of a statistical life” (VSL). Current US regulatory agency practice uses a VSL estimate of roughly $9 million to value an averted death, with a range of empirical estimates of approximately $7 million to $11 million [32,33,34]. That compares with US human capital estimates of lifetime productivity of a little over $1 million [35]. Stated preference estimates of the value of preventing as statistical fatality (VPF) in Europe are lower [36].

The traditional differences between CEA/CUA and CBA/BCA studies have begun to blur, although those changes have not yet affected the NBS economic evaluation literature. On the one side, many CUA studies also report estimates of net monetary benefit calculated by using multiple ICER threshold values as estimates of decision makers’ WTP for health gains [37]. From the other side, some CBAs calculate the value of a statistical life year (VSLY) by dividing VSL estimates by numbers of discounted life-years and multiplying them by projected life-years [33]. For example, a VSL of $9 million may imply a VSLY of roughly $400,000 depending on the age group. VSLY or VPF-based estimates substantially exceed conventional WTP estimates for QALYs [27,36].

The calculation of cost-effectiveness or cost-benefit ratios can be divided in four parts. The first and most important component of an economic evaluation is the quantification of health impact. Without effectiveness in terms of better health outcomes with screening, it is impossible to demonstrate cost-effectiveness. Closely related to this is the calculation of the net economic benefits of improved health outcomes, including reduced treatment costs. The third component is relatively straightforward: how much does it cost to implement a policy, e.g., the added costs to laboratories, healthcare systems, and public health authorities to conduct screening, assure its quality, follow up infants, and provide diagnoses. The fourth and final component is to place monetary or utility values on the health outcomes. In this paper, we focus on the first two components of cost-effectiveness, quantifying the gains in health outcomes and the magnitude of avoided costs associated with improvements in health outcomes.

### 1.2. Assessing Effectiveness in Newborn Screening

To calculate the magnitude of differences in health outcomes that can be attributed to screening requires the assumption of a counterfactual scenario in which the same level of clinical care, including treatment options, are provided for children with and without screening once diagnosed. Many study designs fall short of providing evidence that addresses this criterion. First, the “natural history” of a disorder, *i.e.*, the prognosis in the absence of treatment, is misleading as a comparison, since the availability of treatment must be the same for NBS and non-NBS cohorts to avoid misattribution. Similarly, the use of historical or geographical controls for whom treatment options may have differed can be misleading because it is difficult to separate the impact of screening from differences in clinical care following diagnosis [38,39].

The basic approach to assess net health impacts of NBS in principle is straightforward: compare health outcomes of affected children of the same ages who differed with regard to the timing and type of diagnosis, but were given the same treatments once diagnosed. Specifically, researchers should seek to produce evidence that early *versus* late diagnosis is associated with markedly better health or developmental outcomes at the same chronological age. This last point is crucial. It is common that infants diagnosed presymptomatically with a genetic disorder based on NBS or family history are healthier at the time of diagnosis than those diagnosed at a later age based on the appearance of symptoms [40,41]. Such findings tell us nothing about the effectiveness of early diagnosis in avoiding the subsequent development of symptoms.

Evidence of effectiveness may come from prospective follow-up of screened and unscreened cohorts for a range of endpoints, which may include survival, avoidance of severe morbidity, and retention of normal neurological function *versus* intellectual disability. However, long-term outcomes in screened cohorts are generally not available when conditions are being considered for inclusion in NBS panels. Instead, researchers may have data on outcomes among a group of children with a disorder who were diagnosed at various ages. Early diagnosis may occur as a result of a positive family history, typically the experience of an affected older sibling, or through prenatal or neonatal screening. Analysts can stratify their data by age of diagnosis to assess outcomes for early *versus* late diagnosis. However, it is important to compare outcomes at the same ages in order to avoid bias from age differences in the progression of symptoms. In particular, if outcomes get worse as children get older, children diagnosed as infants will appear healthier than children diagnosed later, on the basis of symptoms, even if there were no effect of early diagnosis.

Another potentially valuable source of information on the impact of early *versus* late diagnosis is paired sibling cohort studies. Such studies follow cohorts of affected children in which an older sibling was detected based on symptoms and a younger sibling was detected based on testing, usually as a result of the positive family history. One can compare outcomes for the siblings at the same age group. One limitation is small numbers; small differences in absolute magnitude are unlikely to be statistically significant even if large in relative size and clinically important. Investigators who follow conventional or frequentist statistical inference will often dismiss such findings as evidence of no association. However, that is an error of statistical inference. Lack of conclusive evidence of effect is not equivalent to evidence of no effect. All that one can conclude from such an analysis is that it is not possible to precisely estimate the magnitude of effect, if any. It is important to compare new findings with previous findings in terms of the direction and relative magnitudes of association to look for consistency.

It is difficult to reliably ascertain long-term health outcomes for unscreened cohorts. One reason is that in the absence of screening, individuals with a given congenital or genetic condition may not necessarily come to clinical attention. That may happen because in some cases the disorder is subclinical, symptoms are nonspecific, or the condition results in early death without postmortem diagnosis. Another reason is that most conditions detected by NBS are rare; to identify sufficient numbers of cases to assess outcomes may require collecting outcomes data for cohorts based on millions of births, which may be impractical. Furthermore, outcomes for historical cohorts, who did not have access to currently available treatments, typically are worse than expected outcomes with current treatments in the absence of screening [38,39].

Data limitations have important implications for the conduct and interpretation of economic evaluations. On the one hand, extrapolation of data from historical controls to the projection of outcomes in the absence of NBS can substantially overstate the health and economic impacts of NBS. Not only may controls lack access to the same interventions following diagnosis, but trends in health outcomes resulting from improved treatments reduces the magnitude of potential health gains from early detection. On the other hand, lack of long-term follow-up data can lead to the understatement of future health and economic benefits. For example, higher economic productivity resulting from improved child health and nutrition is difficult to model and is often left out of analyses. As a result, estimates of net economic benefit may understate the actual benefits.

## 2. Case Studies

### 2.1. Phenylketonuria (PKU)

PKU is an autosomal recessive disorder which, without treatment, results in intellectual impairment and disability. Prior to the development of dietary treatment for PKU in the 1950s, as many as 95% of individuals with PKU developed severe to profound intellectual disability (with IQ < approximately 50), almost all of whom received residential care [42]. According to a later study, about 95% of untreated individuals with PKU had below normal intelligence, with about 80% in the severely to profoundly affected range [43]. During the mid-1950s, low-phenylalanine dietary treatment was developed and shown to be highly effective in preventing further progression of cognitive decline and to prevent the onset of decline when begun in early infancy among younger siblings of affected children [44,45]. Beginning in the late 1950s, a urine test for PKU was widely used in the United Kingdom to screen infants for PKU during home visits [46].

In 1960, Robert Guthrie developed a highly sensitive and inexpensive semiquantitative bacterial inhibition assay to screen for PKU in DBS that could be used in birthing hospitals. A screening study Guthrie conducted among 3,118 residents of the Newark (New Jersey) State School in 1961 found that 21 had PKU [47]. Following a large-scale pilot screening study in 29 US states, NBS for PKU was quickly adopted in most US states between 1963 and 1967 [46,48]. The rationale was the opportunity to avoid preventable severe disability and provide children and their families with the opportunity of healthy, independent development.

A frequent argument made by advocates of screening newborns for PKU was that it would save taxpayers money by reducing the money spent by states on residential institutions [46]. Subsequently, analysts compared the expected reduction in costs resulting from avoided institutionalization with the cost of screening and treatment [49,50,51,52]. In California, detailed cost calculations from the first 2 years of screening showed that the cost per child with PKU detected was $2500 and the cost of dietary treatment for 10 years was approximately $8000 [49]. In comparison, the expected cost of institutionalization over a 30 year period was estimated to be $162,000, for a cost-savings ratio of 15:1. In Canada, Webb suggested that the cost to diagnose and treat one child with PKU for 5 years was $7000, compared with an expected cost of $250,000 to provide lifetime institutional care, a ratio of 36:1 [51].

Other analyses also concluded that screening for PKU would save money, albeit not as dramatically. Steiner and Smith, using data from Mississippi, concluded that screening and treatment for 7 years would cost $56,000 per child with PKU and the avoided cost of institutional care over a 30-year period would be $77,000 per child, a ratio of 1.4:1 [50]. In addition, the authors calculated a benefit-cost ratio, including gain in lifetime productivity as a benefit, of 2.6:1. Van Pelt and Levy used Massachusetts data on screening for PKU and several other metabolic conditions, and reported a cost-savings ratio of 1.8:1; they assumed that just 4 of 7 children with PKU would have required lifetime institutional care [52]. Subsequent economic analyses, whether reported as CEAs or CBAs, have also concluded that screening for PKU is cost-saving or cost-beneficial because of its prevention of severe disability [53,54,55,56,57,58,59,60,61]. For example, in a 2005 CEA study, Geehoed *et al.* projected that 64% of children with PKU would experience severe intellectual disability in the absence of NBS, citing two studies reporting data on children or adults with untreated PKU born in the 1950s or earlier [61].

CBAs of PKU screening have relied on the “human capital” approach to estimating the monetary value of avoided death or disability as the discounted present value of the stream of future annual earnings or productivity in addition to avoided costs of institutional care. According to the “friction cost” approach there is no loss of productivity attributable to congenital conditions [30]. Economic analyses of the expected benefits of screening for PKU, although they appear to have been persuasive, were not based on counterfactual comparisons of screened and unscreened cohorts exposed to dietary treatment. Analysts assumed that the natural history of untreated PKU was the appropriate comparison. They therefore used case series of untreated individuals with PKU as the comparison with cohorts of screened children with PKU. It was widely assumed that children with PKU who are not treated soon after birth would develop irreversible severe cognitive impairment and require lifetime institutional care [46].

However, published data available in the late 1960s and early 1970s belied the assumption that late-diagnosed, late-treated children with PKU have the same prognosis as untreated individuals. Specifically, peer-reviewed studies found that many late-treated children had cognitive test scores either in the low-normal range or had scores indicative of mild intellectual disability [62]. For example, two studies published in 1968 both reported that US or UK children who were put on a low-phenylalanine diet after 4–6 months of age had mean IQ scores of 69 or 77, respectively [63,64].

Experts on PKU came to realize that early cognitive deficits in late-diagnosed PKU with prolonged treatment can be partially reversed in many cases [65,66]. In California, adults with PKU who were born after 1965, but were not detected through NBS, had mean IQ scores of 76 if diagnosed at 3–7 years of age, 92 if diagnosed at 1–2 years of age, and 96 if diagnosed and treated at any time in infancy [62,65]. Despite this recognition among PKU clinical specialists, the NBS community and policy analysts continue to cite obsolete estimates of economic benefits that were predicated on the invalid assumption that late treatment is equivalent to no treatment. A CEA study published in a major peer-reviewed journal in 2006 assumed that in the absence of NBS, 95% of children with PKU would experience moderate to severe developmental delay [60].

Screening for PKU may be less likely to be cost saving (in terms of direct costs) than was previously calculated for a few other reasons [16,67]. First, classical PKU is now recognized to be the severe portion of a spectrum of hyperphenylalaninemia, and a large percentage of infants detected as abnormal by the Guthrie test have mild hyperphenylalaninemia and do not benefit from treatment [68]. Second, the per-person cost of treatment for PKU is now much greater than was assumed previously, when it was thought that older children could safely discontinue the unpleasant, arduous, and expensive low-phenylalanine diet. Since the early 1980s, it has been recommended by experts that dietary therapy be pursued for life [46]. Third, individuals with intellectual disability are now much less likely to be institutionalized than was the case historically, resulting in substantially lower direct costs of care. [69,70]. Fourth, children born to mothers with inadequately treated PKU (maternal PKU) are at risk for birth defects and disability. With NBS, more women with PKU have offspring at risk of maternal PKU and the associated costs of lifetime care [71].

On the other hand, the full benefits to society of screening newborns for PKU in avoiding disability and promoting optimal human development could be even larger than previously estimated. In particular, the economic benefits from improved labor productivity due to gains in cognitive ability are large, even for those who would not be classified as disabled. It has been estimated that each 1 IQ point gain raises lifetime earnings by thousands of dollars [72]. Similar methods have been used to evaluate the economic benefit of prevention of iodine deficiency from the societal perspective [73]. However, direct stated preference estimates of WTP to avoid a 6-point loss of IQ in a child are much smaller than the human capital estimates based on expected gains in lifetime earnings [74].

Studies are also needed to quantify other impacts of prompt *versus* late treatment of PKU such as psychosocial health impacts that can be quantified in terms of QALYs. CUAs of other NBS conditions that result in neurodevelopmental disability have adopted widely varying estimates of utility weights for the calculation of QALY gains from prevention of neurological problems, which calls into question the reliability of the QALY estimates [23].

### 2.2. Cystic Fibrosis (CF)

#### 2.2.1. Health Outcomes

Cystic fibrosis is an autosomal recessive disorder caused by mutations in the *CFTR* gene that is most common in populations of European ancestry. It is a multisystem disease that primarily affects the gastrointestinal and respiratory systems and if not treated typically causes death in childhood from progressive lung disease following recurrent bacterial infections with organisms such as *Pseudomonas aeruginosa*. Approximately 15%–20% of newborns with CF have meconium ileus (MI), an intestinal obstruction present at birth that generally requires surgery to correct and is typically associated with worse outcomes. Most individuals with CF develop pancreatic insufficiency which can cause malnutrition and growth failure.

With improved treatments, most notably in diet, pancreatic enzymes, and nutritional management as well as antibiotic treatments, survival has increased dramatically in high-income countries [75]. For example, median predicted survival increased between 1986 and 2008 from 20.1 to 35.2 years in the Republic of Ireland and from 26.7 to 37.4 years in the United States [76]. In Canada, using a different method, median survival age was calculated to have increased from 31.9 years in 1990 to 49.7 years in 2012 [77]. In Australia, yet another measure, mean age at death, increased from 13.3 years in 1979 to 26.6 years in 2005 [78]. There appear to be differences across countries in CF survival, but it is difficult to compare because of the calculation of non-comparable measures [79]. Less dramatic improvements in lung function have also been reported [80].

Screening newborns for CF using DBS was first implemented in the early 1980s in New Zealand, and portions of Australia, the United States, France, and Italy. A meeting held by the US CF Foundation in 1983 concluded that there was insufficient evidence to warrant screening [81]. Two randomized controlled trials (RCTs) of CF NBS were initiated in the mid-1980s, one in Wisconsin in the United States and one in the United Kingdom, the only such trials of NBS that have been conducted for any NBS disorder [82]. It is unlikely that more RCTs of NBS tests will be conducted in the future. Each of the RCTs had limitations. The published analysis of the UK study [83] had incomplete ascertainment of unscreened children [84] and was excluded from a Cochrane review [85]. The Wisconsin RCT also had disadvantages, including unmatched study arms and the possible alteration of health outcomes in the non-NBS arm due to close clinical monitoring, both of which likely biased comparisons to the null, *i.e.*, no difference in outcomes [86,87].

The Wisconsin RCT yielded evidence of nutritional and growth benefits [85,86], although the lower quality UK RCT did not [83]. Observational studies were also conducted in several countries where screening had been adopted in some places and not others. In 1996, an expert workshop convened by the US Centers for Disease Control and Prevention (CDC) and the US CF Foundation concluded that although there was RCT evidence of nutritional benefit more evidence was needed and called for collection and analysis of additional data, including pilot studies with research protocols [88].

Between 1998 and 2003, several US states started routine screening for CF; one of which, Massachusetts, added screening for CF with parental consent. The British government made a political decision in 2001 to start screening for CF in England and Wales, despite an unfavorable commissioned evidence review [3]. France made a decision in 2002 to screen all newborns for CF, with parental consent, but did not commission an evidence review until years later [89]. The Netherlands took a different approach. A Health Council of The Netherlands systematic evidence review on proposed NBS conditions released in 2005 concluded that screening for CF would be of borderline benefit and called for additional studies [90]; the decision to adopt CF NBS followed in 2010 [91].

The CDC and CF Foundation held another expert workshop on CF NBS in 2003. The result of that meeting and a subsequent evidence review was that there was now sufficient evidence of “moderate” benefit to justify adding CF to NBS programs [84]. Analyses of outcomes of CF NBS generally exclude children with MI from both screened and unscreened cohorts. The strongest evidence of benefit was in improved nutritional status (growth) following the use of pancreatic enzyme supplements and close attention to feeding practices. Two other patient-oriented outcomes were also considered to have fairly strong evidence: improved child survival to age 10 years and better cognitive development among the subset of children at nutritional risk. The CDC report concluded that no consistent evidence of benefit had yet been established for other CF outcomes, including lung function, respiratory infections, health-related quality of life, as well as use and costs of medical care [84].

CF was subsequently added to a recommended uniform screening panel that was adopted by a US advisory committee in 2005 [92]. By 2009 all US states had implemented screening for CF. Canadian provinces followed the US lead beginning in 2007 and by 2015 all but one province, Quebec, screened for CF [1,93,94]. In contrast, in 2011 just 9 countries in Europe screened for CF nationwide, compared with 33 countries screening for PKU [2], an increase from just 2 countries in 2004 [95].

One reason for the relatively uneven adoption of CF NBS in high-income countries, compared with PKU, is the relatively modest benefit from early detection of CF. Until very recently, CF therapies generally only slowed the rate of decline in function rather than restoring normal function. Children with CF detected by NBS typically develop recurrent lung infections and progressive lung disease beginning in early infancy [96,97]. Furthermore, there is a lack of documented evidence that survival or lung function are better in countries with CF NBS than in those without screening. In comparison, differences in treatment practices across countries and centers unrelated to NBS can result in large differences in the magnitudes of clinical outcomes in CF [98,99].

In particular, evidence of improved lung function in cohorts of children with CF detected by NBS is equivocal, as noted above [84]. This is in spite of consistent evidence of improved growth with NBS and evidence that better nutritional status in children with CF predicts better lung function as well as survival [100,101,102], but nutrition is just one of many factors affecting lung function [103]. Neither of the two RCTs of CF NBS found evidence of pulmonary benefit [39,83,84,85]. Children in the NBS arm of the Wisconsin RCT had higher rates of *P. aeruginosa* infection because of earlier exposure to older patients with CF until care protocols were modified [103].

Evidence from observational studies on pulmonary outcomes in relation to age and type of diagnosis is mixed and subject to potential biases. An Australian study that used historical controls born during the years prior to the introduction of NBS reported better lung function in a NBS cohort [104,105], although the use of historical controls has the disadvantage of potential bias resulting from temporal changes in standards of care [39]. A small non-DBS screening study in The Netherlands during 1973–1979 found less decline in lung function in contemporaneous screened children [106], but at least two other studies in different European populations did not find differences [107,108]. One US study that compared children in the same state who were born in hospitals that either did or did not screen for CF found that lung function was initially similar between the NBS and non-NBS cohorts but diverged over time different in favor of the NBS cohort and became significant by age 15 years [109]. One sibling comparison study published in 1977 found significantly better lung function in screened children [110]. Three later sibling studies did not find statistically significant differences in childhood [111,112,113], but one of the studies did find a significant difference in adults [113].

Several analyses of data from the US CF Foundation Registry (CFFR) have reported significantly improved lung function for children with diagnosis through NBS compared to those detected symptomatically [40,80,98]. However, these findings may be a statistical artifact of how diagnosis was assigned in the registry. As this author has previously pointed out, the CFFR classifies all children who were symptomatic (excluding MI) at the time of diagnosis as diagnosed based on symptoms, even if they had been detected by NBS prior to diagnosis [39]. The implication is that children detected by NBS who are symptomatic at birth are assigned by the CFFR to the symptomatic detection group rather than to the NBS group. The exclusion of symptomatic children from the NBS diagnosis group in the CFFR could make the NBS group appear to have better outcomes even if there were no causal effect of early diagnosis. That hypothesis is consistent with the finding in one study that children detected as a result of prenatal diagnosis—none of whom were assigned in the CFFR to the symptomatic diagnosis group—were found to have no significant advantage in lung function, unlike the NBS group [40].

As noted above, one of the most salient potential benefits of CF NBS from a population health perspective is improved survival [84]. Mortality reductions can be modeled in either absolute or relative terms. Formerly, child mortality was common in CF, and older studies often reported large absolute differences in survival with NBS. A meta-analysis of non-US studies reported cumulative death rates by age 10 years of 0.6% in screened and 9.6% in unscreened cohorts [87]. That meta-analysis included data from a follow-up study to UK RCT in which investigators reviewed registry and death certificate data to identify CF-related deaths up to age 5 years, including among unscreened children who were not ascertained in the original study. No deaths were reported among 78 children in the screened group without MI compared with 4 (5.6%) CF-related deaths before 5 years of age among 71 unscreened children without MI (*p* < 0.05) [114].

Sharp drops in child mortality with improvements in CF treatments in recent decades have greatly reduced the number of deaths that can potentially be avoided through early detection by NBS [115]. For example, the Wisconsin trial reported no deaths below age 10 years among the small numbers of enrolled children who did not have MI [87]. A state-level analysis of CFFR data for survival among children with CF born during 1986–1991 found a 1.7 percentage point difference in mortality through age 9 years in states with and without CF NBS, 0.65% *versus* 2.35%, or a relative reduction of 72% [87]. That finding was not statistically significant but is consistent with improved survival, albeit not precisely estimated. The authors acknowledged that differences in quality of care between states might have contributed to the difference between states in CF child deaths. An individual-level analysis of CFFR data also reported that children detected in the first month of life without MI had significantly improved survival regardless of whether they were classified with screening (NBS or prenatal) or symptomatic diagnosis [116].

One study suggests that a survival advantage of CF NBS may extend into adulthood [105], although this requires replication. In the Australian historical cohort study discussed above, a statistically significant survival advantage at 10 years, which was attenuated at age 15 years [87], became stronger at 25 years follow-up [105]. Specifically, 61% of the pre-NBS cohort had either died or undergone lung transplant by age 25, compared with 34% of the NBS cohort. Both cohorts had relatively unfavorable outcomes compared with a Dutch study of 52 sibling pairs with CF which reported that 3 older siblings *versus* 1 younger sibling died prior to age 25 and 2 *vs.* 0 underwent lung transplants [113]. Slieker *et al.* concluded that a p value of 0.21 for the first comparison indicated “no differences” in survival [113]. However, the absence of a statistically significant difference is not evidence of no difference; the findings are consistent with a large relative reduction in mortality with early detection.

#### 2.2.2. Economic Evaluations

Published or publicly disseminated systematic evidence reviews and health technology assessments of adding CF to NBS panels did not include estimates of cost-effectiveness owing to a lack of published full CEAs at the time reviews were prepared. The Alberta HTA program in 2007 undertook a review of published economic analyses of CF NBS and prepared their own calculations of the cost of implementation [117]. The Washington State Department of Health constructed a CBA model of CF NBS in 2004–2005 which projected a benefit-cost ratio of at least 4 to 1, assuming a child mortality reduction of 1–2 percentage points [118] that was consistent with subsequently published US estimates [87]. That CBA was essential to the policy decision in Washington State to add CF to their NBS panel. [16] Although that analysis was never officially released, it is discussed in a forthcoming paper.

Three full CEAs of NBS for CF have been published in English [119,120,121], along with one partial CEA [122] that calculated net costs but did not quantify health outcomes. Several other English-language partial economic evaluations of CF NBS have been published. Two decision analyses compared the costs associated with different NBS protocols to identify the most efficient screening strategies [123,124]. Two other studies assessed costs associated with CF NBS and diagnostic tests in Wisconsin [125,126]. All four of the CEA studies estimated that at least one screening strategy would be cost-effective relative to no screening [119,120,121,122]. However, there were disagreements among the studies as to which screening strategy would be most cost-effective, what outcomes would be improved, and by how much treatment cost would be reduced.

A recent cost accounting study from Wisconsin estimated the total added cost of CF NBS, including diagnostic testing, be about $7 per infant tested for an algorithm using molecular genetic testing as a second-tier screen [123]. The Dutch CEA studies assumed similar incremental costs of screening, somewhat lower for strategies not using molecular testing largely because of the high assumed cost of genetic counseling [121].

The first CEA study, from the United Kingdom, was the only one which projected QALY gains rather than life-years gained. The study optimistically assumed that screening would delay the onset and progression of CF respiratory symptoms by an average of 6 months, thereby resulting in better lung function and health-related quality of life, modeled based on lung function [119]. These assumptions were adopted despite the authors’ acknowledgement of a lack of supporting evidence. In addition, it was assumed that screening would sharply reduce costs of treatment. On the basis of those optimistic hypothetical assumptions, including a relatively low cost of screening, it was calculated that screening would be highly cost-effective, with an ICER <£7000 per QALY gained [119]. To calculate QALY gains from NBS Simpson *et al.* [119] compared an average utility weight of 0.75 for a sample of patients with symptomatic CF to an arbitrary value of 0.95 for asymptomatic patients. However, the internal comparison of data for UK patients with CF showed relatively small differences in utility weights for patients with varying levels of lung function [127].

Two subsequently published CEA studies assumed improved survival with NBS but no differences in treatment costs, lung function or health-related quality of life. The authors of the two studies assumed 25% relative and 1.5% absolute reductions in cumulative child mortality with 94% of infants with CF assumed to survive to age 6 years in the absence of NBS [120,121]. Both studies projected that certain screening approaches for CF would likely have an ICER < €30,000 per life-year gained and would therefore be considered cost-effective. If treatment costs were reduced with early diagnosis, it was concluded that screening might even be cost-saving [121]. However, the absolute mortality assumption in the two studies does not appear to be consistent with data from The Netherlands. According to registry data, roughly 98% of infants with CF born in The Netherlands during 1990–1994 survived to age 6 years, without NBS [128]. Consequently, the validity of the ICER estimates of the two studies is unclear.

One partial CEA study from Quebec, Canada, calculated total costs and numbers of cases of CF detected, and projected that total costs would be lower with NBS. The authors stated that screening dominates no screening, *i.e.*, is cost-saving with better health outcomes, although they did not calculate health outcomes. Nshimyumukiza *et al.* assumed, based on an unpublished analysis of provincial hospital statistics, that average costs of hospital care would be almost 85% lower for children identified through NBS [122]. In contrast, the Washington CBA study assumed a more modest reduction of one hospitalization per child detected by NBS, equivalent to a roughly 25% lower hospitalization cost [118].

Several studies have reported reduced costs of hospital care with CF NBS [95]. First, the published analysis of data from the UK RCT reported 30% fewer hospital days during infancy for infants detected by screening than infants born in the same area who were diagnosed clinically [83]. However, because there was incomplete ascertainment of cases in the clinical cohort and infants with mild cases of CF were likely not ascertained, the comparison may be biased in favor of the NBS cohort. More importantly, the higher quality Wisconsin RCT, which had complete ascertainment and follow-up, found no difference in hospital costs for children with CF in the two study arms [123,126].

Findings from observational data, unlike the Wisconsin RCT, indicate lower hospitalization costs in CF NBS cohorts. The Australian historical control study and the French study that compared two neighboring regions both reported significantly fewer hospitalizations in the NBS cohorts [108,129]. Further, a UK registry study found that average treatment costs were lower by 21%–35% for children aged 4–9 years who were diagnosed through NBS in Scotland than those living in England, which at the time did not have a country-wide CF screening program [130]. However, geographic comparisons do not control for potential differences in care practices between regions with and without NBS programs. An analysis of individual-level US CFFR data also reported that fewer children classified as diagnosed by NBS were hospitalized in infancy [40], but as noted above infants detected through NBS who were symptomatic were excluded from the NBS group.

The cost-effectiveness of NBS for CF is also influenced by other economic assumptions. One is the avoidance of costs associated with the “diagnostic odyssey” (repeated examinations and laboratory tests and acute care visits to treat symptoms) entailed in reaching a definitive diagnosis of CF in the absence of NBS. Two CEAs assumed that reduced diagnostic costs would offset 16%–36% of the cost of screening [119,120]. However, estimates of the healthcare costs associated with CF diagnosis in the absence of NBS are variable. An audit of UK costs for 25 children with CF suggests cost < £1000 [119]. A more comprehensive audit of 36 Dutch patients yielded an equivalent cost estimate of approximately €9000 [120].

The first Dutch CEA calculated through a sensitivity analysis that two additional assumptions could make screening cost-saving (negative total cost). [120] First, they calculated that a reduction in the ordering of sweat tests, similar to that which was reported in Wisconsin following the introduction of screening for CF [125], could offset almost 40% of the added cost of screening. Second, if NBS were to lead to 30% fewer births affected by CF, NBS for CF would be cost-saving. However, preliminary findings from France of a lower birth prevalence of CF attributed to NBS [131] were not supported by a subsequent report, nor by subsequent studies from other countries [132,133].

The recent Dutch CEA incorporated the assumption of a large reduction in ordering of sweat tests into the base-case model rather than as a sensitivity analysis [121]. This assumption was crucial to their conclusion that CFS NBS would be cost-effective, with an ICER < €30,000 per life-year gained. If no reduction in sweat tests was assumed to occur, the ICER would be approximately €75,000 per life-year gained and screening would not be considered cost-effective. Conversely, if the number of sweat tests decreased by 90% with NBS, other things constant, screening would likely be cost-saving [121].

## 3. Discussion

Estimates of cost savings or cost-effectiveness of NBS are necessarily dependent on quantitative estimates of effectiveness derived from clinical and epidemiologic research. Screening per se does not improve outcomes; it is the interventions that are enabled by early diagnosis that alter outcomes. Therefore, cost-effectiveness is crucially a function of the effectiveness of available therapies. An obvious difference between PKU and CF is that PKU has a highly effective therapy, which can virtually eliminate the most important adverse health outcome of the untreated disorder, whereas CF currently does not. An important determinant of the reliability or robustness of CEA estimates is the quality and consistency of epidemiologic evidence of effectiveness [134,135]. Although there is a high confidence in the nutritional benefits of CF NBS, estimates for other outcomes are less certain. Given that ICER estimates rely on assumptions of non-nutritional benefits, including reductions in diagnostic and treatment costs as well as survival, the robustness of published CEA estimates for CF NBS can be considered to be relatively low. The implication is that more research is needed to provide more robust estimates of outcomes of CF NBS, in particular survival and hospitalization costs, to inform future economic evaluation studies.

Cost-effectiveness is also crucially dependent on the extent to which the therapeutic effectiveness varies with the age at which treatment is begun. If a disorder has a therapy that is effective when it is initiated, even if the diagnosis is delayed, the effectiveness and cost-effectiveness of NBS will be lower than if it is essential for the treatment to be administered during a presymptomatic period in order to be effective. Demonstrating positive outcomes through long-term follow-up of a screened cohort is not sufficient to quantify the effectiveness of NBS and early treatment; one must also have comparable long-term outcome data for late, symptomatically diagnosed individuals who received standard-of-care treatment once diagnosed. It is the difference in the outcomes of the two groups that determines the magnitude of effectiveness of early detection.

When NBS for PKU was established, it was believed that in order to avoid severe, irreversible intellectual disability it is essential to begin dietary therapy within the first months of life. That, combined with the practice at the time of routine institutionalization of persons with intellectual disability, made the economic argument for NBS very compelling. Even though there is now good evidence that progressive cognitive deterioration in children with unscreened PKU can be halted and in many cases even partially reversed with dietary treatment following diagnosis [62], the value proposition for PKU NBS is still highly compelling. Early initiation of treatment and maintenance of effective dietary control of blood phenylalanine following NBS has been shown to make a large difference in ultimate cognitive and behavioral outcomes for individuals with PKU, although research on newer, more effective treatments is ongoing [136]. However, outcomes in many cases are not optimized, and barriers to accessing treatments for adults with PKU remain a serious issue [137].

Previously calculated economic benefits of PKU NBS may now be substantially lower, owing to different patterns of care, the partial reversibility of cognitive impairment in many cases, even with late initiation of dietary treatment, and higher costs of dietary treatment. On the other hand, economic benefits that were not previously taken into account, such as impacts of reduced cognitive ability short of overt disability on economic productivity, could be included. Updated estimates of the magnitude of cost savings and benefit-cost ratios for PKU NBS are needed. However, it is challenging to quantify the number of IQ points gained per individual with PKU, as well as the monetary value of an IQ point, and it is even more challenging to measure and value other endpoints, such as executive function.

One factor that could potentially affect calculations of the cost-effectiveness of NBS for condition such as CF is consideration of the impact of the detection of ambiguous cases or cases that may develop clinical symptoms at some point years in the future. Infants with abnormal CF screening results and inconclusive confirmatory testing results are referred to as having “CFTR-related metabolic syndrome” or CF screen positive, inconclusive diagnosis” (CFSPID) [138]. Indeterminate or equivocal diagnoses can impose costs on the healthcare system and families, which have not been estimated or included in any of the published CEAs. Research is needed to understand the long-term health and economic consequences of the clinical follow-up of children with equivocal diagnoses.

Quantifying the cost-effectiveness and cost-benefit of screening for CF is particularly challenging, despite a large number of empirical studies reporting long-term outcomes in both screened and unscreened cohorts. The nutritional benefits of early detection and treatment of CF are well established, but it is hard to quantify the direct benefits of better nutritional status. Higher percentiles of height-for-age and weight-for-age have been shown to predict better lung function as children age [100]. That might account for the finding reported in some studies that children with CF detected by NBS had significantly less deterioration in lung function over time [106,109]. Better nutritional status has also been reported to predict better health-related quality of life among children ages 9-19 years participating in the Wisconsin study [139].

More research is needed to better quantify the long-term health benefits of CF. One challenge is that since child mortality in CF is now rare in high-income countries, it is less salient and much harder to estimate with precision. Nonetheless, it is important to confirm previous US estimates based on comparisons of 10-year CF survival rates for states that were or were not screening for CF prior to 1996 [87] using more recent data to assess outcomes in states that began screening between 1998 and 2003. In addition, fixed effects statistical analyses that compare hospitalization costs in US states or Canadian provinces for infants and young children with CF based on the timing of adoption of CF NBS and controlling for pre-existing geographic differences in care patterns could be informative.

The quantification of the health benefits of NBS for specific disorders is desirable. However, in many countries, including the United States, decisions on whether to add disorders to NBS panels appear to be primarily qualitative, based on evidence of avoidance of severe morbidity or mortality along with considerations of perceived test accuracy, feasibility, and affordability [16]. The US Advisory Committee on Heritable Disorders in Newborns and Children currently contracts evidence reviews for candidate disorders [140]. As part of that process, decision analytic models are set up to quantify expected health outcomes [141]. However, the published evidence-base for candidate disorders is often insufficient to support meta-analyses of health outcomes. In any case, the decision matrix used by the Committee to make recommendations is qualitative. The historical case studies of the US adoption of PKU and CF NBS, both of which predated the current decision-making matrix, are also consistent with the dominance of qualitative assessments of clinical benefit in the decision-making process for NBS panels.

In jurisdictions in which health policy decisions are based in part on explicit considerations of cost-effectiveness, the quantification of effectiveness may be more critical. However, the limited salience of the quantification of benefits and cost-effectiveness in NBS decision making appears to be true in many other countries, even those in which cost-effectiveness is officially listed as a criterion. An analysis of 22 NBS expansion decisions in European Union member countries found that just two (9%) were accompanied by quantitative meta-analysis of evidence of benefit and just four (18%) were informed by CEAs [17]. Three of the 22 decisions were made by the UK National Screening Committee, for CF, sickle cell disease, and medium-chain acyl-CoA dehydrogenase deficiency screening in England [142]. Previous publications have discussed the policy decisions for CF and sickle cell disease screening in England, including the limited influence of CEA calculations [143,144].

## 4. Conclusions

In conclusion, attempts to assess the effectiveness of NBS in improving health outcomes constitute a critically important part of the process for deciding which conditions should be added to NBS panels. Such assessments can be both quantitative and qualitative. If information is available to quantify both health outcomes and short-term and long-term costs, economic evaluations can also be undertaken even if cost-effectiveness or cost-benefit are not treated as formal criteria for expansion of NBS panels. Such analyses should be regarded as preliminary in nature and should be revisited in the future once more complete information becomes available. Although that would be too late to inform policy decisions on adding NBS disorders, the information might be of use to other jurisdictions considering NBS expansions.
